# Uptake routes of microplastics in fishes: practical and theoretical approaches to test existing theories

**DOI:** 10.1038/s41598-020-60630-1

**Published:** 2020-03-03

**Authors:** S. Roch, C. Friedrich, A. Brinker

**Affiliations:** 1 Fisheries Research Station Baden-Württemberg, Argenweg 50/1, 88085 Langenargen, Germany; 2 University of Konstanz, Mainaustraße 252, 78464 Konstanz, Germany; 3 FMF Freiburg Material Research Centre and Institute for Macromolecular Chemistry, University of Freiburg, Stefan-Meier-Straße 21, 79104 Freiburg i. Br., Germany

**Keywords:** Freshwater ecology, Environmental impact

## Abstract

Microplastics are frequently detected in the gastrointestinal tracts of aquatic organisms worldwide. A number of active and passive pathways have been suggested for fish, including the confusion of microplastic particles with prey, accidental uptake while foraging and transfer through the food chain, but a holistic understanding of influencing factors is still lacking. The aim of the study was to investigate frequently suggested theories and identify relevant biotic factors, as well as certain plastic properties, affecting microplastic intake in fish. Four species of freshwater fish, each representing a different combination of foraging style (visual/chemosensory) and domestic status (wild/farmed) were exposed to different realistic plastic concentrations and polymer types with and without the provision of genuine food. As most previous investigations of microplastic uptake routes consider only particles large enough to be perceptible to fish, the potential for accidental intake via drinking water has been somewhat neglected. This route is evaluated in the current study using a model approach. The results show that visually oriented fish forage actively on microplastic particles that optically resemble their usual food, while fish with a predominantly chemosensory foraging style are more able to discriminate inedible food items. Even so, the accidental uptake of microplastics while foraging is shown to be relevant pathway, occurring frequently in both visual and chemosensory foragers alike. Several factors were shown to increase plastic uptake, including microplastic concentration in the water, foraging behaviour promoted by availability of genuine food, and fish size. Although both wild and farmed fish ingested microplastic particles, cultured fish showed less discernment in terms of colour and were more likely to forage actively on microplastics when no food was available. Drinking has been identified as a possible source of microplastic intake specifically for large marine fish species. Particles smaller than <5 µm can pass the gastrointestinal tract wall and bioaccumulation could arise when uptake exceeds release or when particles are assimilated in tissues or organs. The effects of accumulation may be significant, especially in long-living species, with implications for food web transfer and fish as food items.

## Introduction

One of the first articles published on the subject of microplastics in the aquatic environment described the presence of plastic spherules in fish^1^. Since then, the ingestion of microplastics by aquatic organisms has been recognized as one of the major detrimental effects of plastic pollution worldwide^2^. Microplastics are mostly defined as synthetic polymer particles and fibres smaller than 5 mm^3^ and numerous studies suggest that they affect virtually all marine and freshwater fauna^4,5^. The origins of microplastics can be diverse, but most have been shown to derive from terrestrial sources^6^.

**Figure 1:**
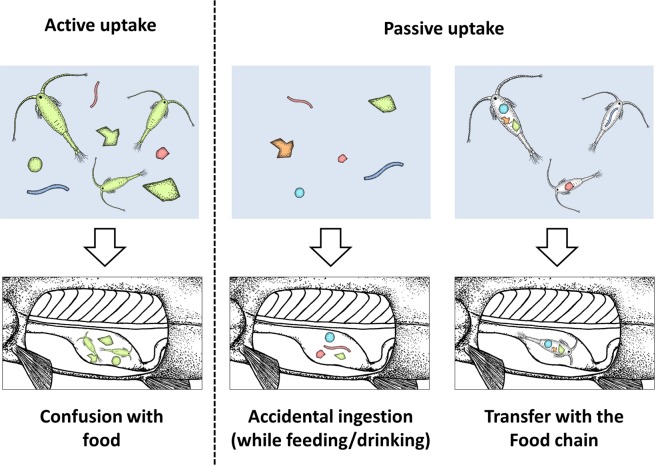
Possible uptakes routes of microplastics in fish.

Despite their ubiquity in aquatic organisms, knowledge of the uptake routes of microplastics remains fragmentary^7^. For fish, several pathways have been suggested (Fig. 1). Firstly plastic particles may be deliberately ingested having been mistaken for food. There is evidence that some fish species actively forage on microplastics that visually resemble their prey in some way^8,9^. Second, microplastics might be ingested passively or accidentally while foraging, and thirdly they may be transferred via the food chain^10,11^. Several examples of the latter have been described, showing the potential transfer of microplastics from prey to predator^12–14^. However, a holistic understanding of all active and passive uptake routes is still lacking, controlled laboratory experiments are scarce and available studies do not generally consider factors such as habitat and related feeding preferences, the effects of particle concentration in the water or the genetic origin of fish.

Most fish species have well developed eyes^15^ and most planktivorous and piscivorous species rely on visual cues while foraging^16,17^. On the other hand, most mainly benthic fish species, such as cyprinids, also possess a well-developed sense of taste, which allows them to distinguish edible and inedible food items they cannot easily see^18^. This chemosensory ability is especially important when searching for food on or directly in the sediment of a river-, lake- or seabed^19^. Previous analyses of field data have not yet resolved possible influences of fish habitat preference or of related feeding preferences on microplastic uptake^20–22^. Nevertheless, it is assumed that chemosensory foragers should be better equipped to discriminate non-edible particles than visual foragers^23^. In this context, despite the globally practiced management technique of stocking and the problem of escapees^24,25^, the potential effect of domestication on food selection behaviour in fish is rarely considered^26–28^. In freshwater environments especially, billions of fish are stocked every year to conserve or restore natural species assemblages or to increase the abundance of favoured species^24^. It is suggested that the age and experience of released fish and their origins from domesticated or wild lines, may alter or reduce their ability to discriminate plastic debris as non-food.

Another previously neglected uptake route could be the ingestion of very small particles along with water. While drinking rate in freshwater fish species is comparatively low, marine fish drink almost continuously in order to maintain homeostasis^29^. Thus in marine environments especially, very small particles might be passively ingested on a regular basis.

The aim of this work was to generally test the above-mentioned uptake routes (Fig. 1) in a laboratory setup and to examine the importance of relevant biotic factors and certain plastic properties on microplastic intake. Four fish species were selected, each representing a different combination of foraging style (visual/chemosensory) and genetic status (wild/domestic). Fish were exposed to three environmentally relevant plastic concentrations and commonly used polymer types with and without the provision of genuine food. In addition to previously investigated uptake routes, the hitherto unconsidered potential for small particles to be imbibed with drinking water was theoretically evaluated. To quantify the passive uptake of microplastics, a model was developed which took into account both uptake and egestion. Besides uptake rates and accumulation effects, the potential for long-term accumulation in tissues and organs was also considered. The results of the practical and theoretical approaches are discussed and their relevance for the field is evaluated.

## Methods

### Husbandry of experimental fish

Four representative fish species were selected to examine the effect of domestication (wild vs cultured) and foraging style (visual vs chemosensory) on microplastic uptake. The species were: rainbow trout (*Oncorhynchus mykiss*), grayling (*Thymallus thymallus*), common carp (*Cyprinus carpio*), and crucian carp (*Carassius carassius*). The rainbow trout and common carp originated from cultured lineages bred specifically for aquaculture, while the grayling and crucian carp were raised from wild offspring. All experimental fish were acclimatized for two weeks before the exposure experiments began. Fish were held in tanks (50 fish per tank, tank size: 0.5 × 0.55 × 0.55 m) and fed twice a day, six times a week with commercial pellet food (rainbow trout, grayling: Inicio 702 (2 mm), Biomar, Denmark; common carp, crucian carp: Vital (2 mm), Alltech Coppens, The Netherlands). An overview of the relevant husbandry parameters is given in Table 1. The experimental tanks were cleaned daily and water parameters were constantly monitored by probes and an electronic surveillance system. All experiments were performed in triplicates.

**Table 1:** Overview of relevant husbandry parameters of experimental fish. ^a^light regime: 12 h illumination with 30 mins dawning periods, ^b^percent of fish body weight.

Fish species	System, exchange rate	Temperature [°C]	Oxygen [mg/L]	Light intensity^a^ [Lux]	Feeding quantity [%]^b^
rainbow trout, grayling	flow-through, 8 L/min	8 ± 0.3	8–10	300	1.5
common carp, crucian carp	semi recirculating aquaculture system, 3 L/min	20 ± 0.5	8–10	300	2.0

### Exposure experiments

To study the effects of plastic colouring, particle density and particle concentration on uptake by fish, fragments of six very widely used plastic polymers^30^ with distinct properties were tested: polyethylene (PE, Falcon tube cap, blue), polypropylene (PP, storage box, grey), polystyrene (PS, plastic pellets, Glow-Side, Kretz, Germany, yellow), expanded polystyrene (EPS, packaging material, white), polyethylene terephthalate (PET, shampoo bottle, clear/purple) and polyvinyl chloride (PVC, water pipe, brown). The plastic was chopped into square particles of approximately 1–2 mm width with the help of a scalpel and stored in tap water for 24 h to minimize hydrophobicity.

During the experiments, 50 individuals of each species were exposed to three environmentally relevant particle concentrations, which were derived from available freshwater studies^31^. The concentrations were: 100, 1000 and 5000 microplastic particles per m^2^, or 0.19, 1.9 and 9.1 particles per litre. Corresponding particle numbers of each polymer type were evenly distributed into the tanks and remained there for two hours. No food was provided during the experiment and subsequently, all particles were removed. Ten fish were sampled from each tank directly after exposure (0 h) and a further ten after 6 and 24 hours respectively. The experiments were then repeated with the same experimental design, but the fish were also provided with genuine food after the plastic particles were added to the water. Feeding was repeated after one hour of exposure (total amount of food: rainbow trout and grayling: 1.5%, common carp and crucian carp: 2.0% of fish body weight). Sampled fish were anesthetized with clove oil (0.1 mL per L water), euthanized with a cut at the gills and stored at −20 °C until required for further examination.

### Detection of microplastic particles in the gastrointestinal tract

Sampled fish were thawed and each individual was weighed to the nearest 0.1 g and measured for total length to the nearest 0.1 cm. The stomach (rainbow trout, grayling) or the whole gastrointestinal tract (common carp, crucian carp) was dissected and placed into a 250 mL glass beaker. To digest the organic matter, 25 mL of sodium hydroxide (NaOH, 1 mol L^−1^, Chemsolute, Th.Geyer, Germany) was added to the beaker and heated to 50 °C on a hot plate for 15 min while being mixed with a stirring bar. Each sample was diluted with 125 ml with filtered ultrapure water (0.2 μm pore size, Arium 611, Sartorius Stedim Biotech, Goettingen, Germany) and vacuum filtered through a 300 µm gauze. Recovered microplastic particles were examined under a dissecting microscope (Zeiss, Stemi SV6, 8−50x magnification) and their number and polymer type was noted. The prevalence of microplastic (percentages of burdened fishes), abundance (number of microplastic particles per fish) and the intensity of microplastic burden in contaminated fish (number of microplastic particles per fish) were calculated.

### Model to estimate passive uptake of microplastics

In addition to the uptake routes tested above, the previously neglected possibility of a microplastic uptake via drinking was assessed. To estimate particle uptake rates, the following model was developed:

1 $${C}_{F}=({Q}_{dr}\cdot {W}_{F})\cdot t\cdot {C}_{E}$$

where *C*_*F*_ is the microplastic intensity in the gastrointestinal tract (GIT) of an individual (number of particles per fish), *Q*_*dr*_ is drinking rate in L per hour and kg fish weight, *W*_*F*_ is fish weight in kg, *t* is exposure time in hours and *C*_*E*_ is microplastic concentration in the water (number of particles per L). The distribution of particles in the water column was assumed to be random.

Three weight classes of fish (0.1 kg, 0.5 kg, 1 kg) were considered and the drinking rates of freshwater and marine fish were derived from the literature (freshwater fish: 0.4 ml kg^−1^ h^−1^, marine fish: 4.1 ml kg^−1^ h^−1^, see Supplementary Table S1 online). Exposure time was set to 1000 h. The microplastic concentration in the water was the same as in the previous exposure experiments at 0.19, 1.9 and 9.1 particles per L.

Equation (1) was extended to examine possible accumulation of microplastics imbibed through constant water uptake in the GIT, with the second term simulating the egestion of the particles over time:

2 $${C}_{F}=({Q}_{dr}\cdot {W}_{F})\cdot t\cdot {C}_{E}-\frac{t}{{t}_{F}}$$

The variable *t*_*F*_ accounts for the average residence time of microplastics in fish and was derived from the literature (mean: 44 h, see Supplementary Table S1 online). The model was computed with the same values as mentioned above.

The final approach evaluated the long-term accumulation of imbibed microplastic particles smaller than 5 µm in the tissue or organs of fish:

3 $${C}_{F}(t)=({Q}_{dr}\cdot {W}_{F})\cdot t\cdot ({C}_{E}\cdot p\cdot q)$$

As only a fraction of microplastic particles are small enough to cross the intestinal barrier, the variable *p* accounts for the percentage of particles in this relevant size range. Only particles smaller than 5 µm are regarded as small enough to be translocated on a regular basis^32,33^. The percentage of particles of relevant size was derived from an extrapolation in a previous study of microplastic burden intensity in freshwater fish, which suggested that 70% of particles in fish might be expected to be sub-5 µm^22^. A particle concentration of 1.9 particles per L in the water column was assumed. The variable *q* considers the percentage of particles smaller than 5 µm actually crossing the intestinal barrier. While no reliable means of estimating the rate of translocation of microplastics into tissues in fish under realistic conditions has yet been reported, it should be noted that the translocation rate is likely to vary according to particle size and other factors. To account for this uncertainty, conservative rates of between 0.01 and 1%, similar to those in rodents, were assumed^33^, though higher values of up to 10% have been reported^34–36^. To account for weight changes and related increases in water uptake, weight for each year was calculated, using a modified von Bertalanffy growth function^37^:

4 $${W}_{F}=a\cdot {{L}_{{\rm{\infty }}}}^{b}\cdot {[1-exp(-K\cdot (t-{t}_{0}))]}^{b}$$

The variables *a* and *b* are length-weight relationship parameters, *L*_*∞*_ is asymptotic length (theoretical maximum length) in cm, *K* is the body growth coefficient and *t*_*0*_ is age of zero weight. All parameters were derived from the website fishbase.org, using common carp and cod (*Gadus morhua*) as representative freshwater and marine fish species respectively. The number of particles accumulated in the tissues and organs for each year of growth up to 15 years was calculated.

### Statistical analysis

To assess the individual effects of biotic and abiotic factors on the microplastic uptake in fish, different linear models were selected by minimizing the Akaike information criterion (AIC)^38^ as follows:

5 $${y}_{i}={\beta }_{0}+{\beta }_{1}{x}_{1}+\ldots +{\beta }_{i}{x}_{i}+{\varepsilon }_{i}$$

where *y*_*i*_ is the dependent variable, *β*_*0*_ is the intercept, *β*_*i*_ is the regression coefficients, *x*_*i*_ is the predictor variables, and *ε*_*i*_ is the random residual error. The following dependent variables were defined: prevalence, abundance and intensity (for definition see above). The utilized models and associated model effects are summarized as Supplementary Table S2 online. To examine relationships between certain relevant factors, Response Surface Methodology (RSM) graphs were plotted based on the respective linear model.

### Ethical Statement

All methods were carried out in accordance with ethical guidelines and regulations. All experiments were conducted according to the German Animal Welfare Act (TierSchG) and approved by Referat Tierschutz of Regierungspräsidium Tübingen (LAZ 2/16, AZ 35/9185.81-4).

## Results

### Biotic factors influencing microplastic uptake

**Figure 2:**
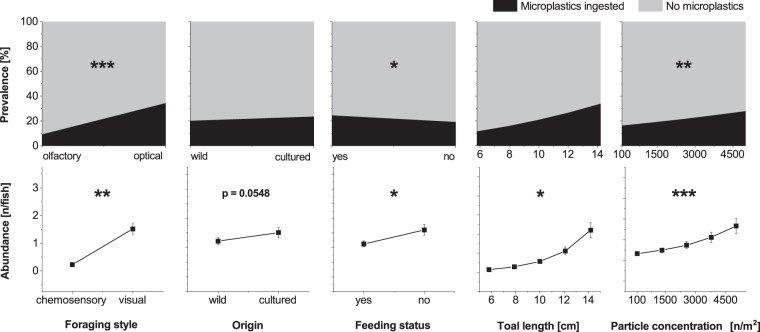
Microplastic uptake (Grand marginal means) directly after exposure. **Top:** Microplastic prevalence, **Bottom:** Microplastic abundance (±standard error). Asterisks indicate statistically significant differences between model effects (*P < 0.05, **P < 0.01, ***P < 0.0001). n = number of particles.

Three out of four fish species, with the exception of crucian carp, ingested microplastics during the exposure experiments. The mean total lengths and fresh weights of sampled fish are summarized as Supplementary Table S3 online. Mean microplastic prevalences and abundances (± standard error) for each fish species and particle concentration over time are summarized as Supplementary Figs. S1 and S2 online. The performed nominal-logistic model (whole model: 720 observations, d.f. = 5, r^2^ (U) = 0.1947, P < 0.0001) revealed significant factors influencing microplastic prevalence directly after exposure (Fig. 2). The main orientation sense used by foraging fish had a highly significant effect, with visual foragers ingesting particles more often than chemosensory foragers (P = 0.00022). When genuine food was available during exposure to microplastics, the portion of fish ingesting microplastics was lower than when real food was unavailable (P = 0.01528). Particle concentration had also a significant effect, with prevalence of burdened fish increasing with concentration (P = 0.00036). On the other hand, the origin and total length of fish didn´t significantly influence microplastic prevalence (P > 0.05).

**Figure 3:**
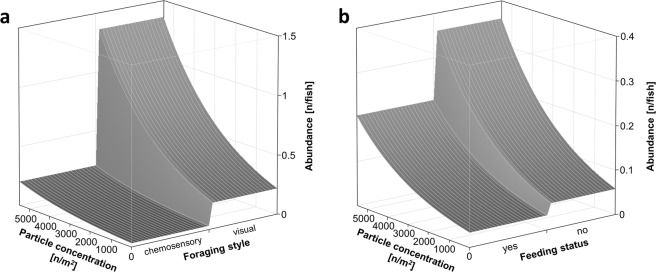
Response surface methodology graphs based on the results of a General Linear Model for particle concentration in water versus **(a)** foraging style and **(b)** availability of genuine feeding opportunity. n = number of particles.

When looking at particle abundance directly after exposure, the General Linear Model (GLM, whole model: 720 observations, d.f. = 5, P < 0.0001) revealed several significant factors, affecting the quantity of microplastic ingested by fish (Fig. 2). Foraging style and particle concentration had an effect similar to that of prevalence (foraging style: P = 0.00033, particle concentration: P < 0.0001), with abundance increasing exponentially with the concentration of microplastics in the water. In contrast, abundance was higher when fish were not provided with genuine food (P = 0.00127). Total body length also had an exponential significant effect on the number of plastic particles ingested (P = 0.00101). A further trend was apparent with fish origin, where wild fish ingested fewer microplastic particles (P = 0.05480) than cultured ones. To explore the relationship between identified relevant biotic factors and particle concentration in the water, RSM graphs were plotted. The results for foraging style and feeding activity versus particle concentration in the water are shown in Fig. 3(a,b). In visual foragers, abundance increased exponentially with the particle concentration in water, whereas there was only a minor increase in chemosensory foragers. Abundance increased exponentially with particle concentration in water, regardless of whether genuine food was supplied or not, though the increase was lower when fish were properly fed.

To test if there was any egestion of microplastics during the exposure experiment, a GLM was performed for different time points (whole model: 1860 observations, d.f. = 6, P < 0.0001). Fish species, active feeding and particle concentration all had a significant effect on particle abundance in exposed fish (fish species: P < 0.0001, feeding status: P < 0.0001, particle concentration: <0.0001), while sampling time had no influence (P > 0.05).

### Particle properties influencing microplastic uptake

**Figure 4:**
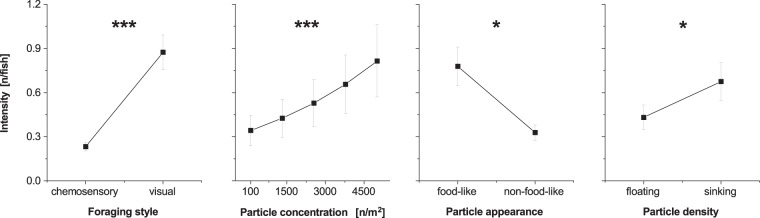
Effect of particle properties on microplastic uptake (± standard error, all sampling points). Asterisks indicate statistically significant differences between variables (*P < 0.05, **P < 0.01, ***P < 0.0001). n = number of particles.

When looking at particle properties, the GLM (whole model: 2616 observations, d.f. = 5, P < 0.0001) revealed several significant factors influencing microplastic intensity (Fig. 4). Time after exposure had no significant effect on the number of particles identified in fish GIT, indicating no bias resulting from microplastic excretion (see above), so all data was included in the model. Particle concentration in the water and foraging style had a significant positive effect on the number of microplastic particles in fish (particle concentration: P < 0.0001, foraging style: P < 0.0001). Furthermore, particle density and particle appearance affected particle numbers (particle density: P = 0.00118, particle appearance: P = 0.04382), with food-like particles and sinking particles being ingested most often. Carp, in contrast to the two visually foraging fish species, ingested solely sinking particles.

**Figure 5:**
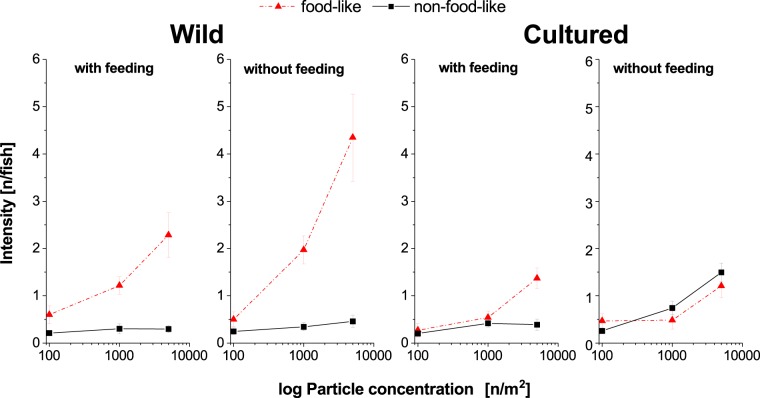
The effect of feeding and particle appearance on particle intensity (mean ± standard error) in visually oriented fishes (all time points). n = number of particles.

To further analyse the interactions between relevant factors identified in the GLM above in visually oriented fish, a generalized regression model was performed (whole model: 2400 observations, d.f. = 5, P < 0.0001). The results are summarized in Table 2. All factors, except the interaction between genetic fish origin and particle concentration significantly affect the intensity of microplastic burden. An overview of the mean particle intensity of food-like and non-food-like particles, dependent on particle concentration, fish origin and feeding status is given in Fig. 5. The corresponding prevalence of ingested food-like particles in visually foraging fish is summarized as Supplementary Fig. S3 online. In wild fish, the intensity of food-like particles increased with particle concentration in the water, independent of whether fish were feeding. In contrast, particles not resembling their food were ingested in low numbers and didn´t increase with particle concentration in the water. In cultured fish, microplastic intensity was generally lower, but when no food was provided, numbers of both food-like and food-unlike particles ingested increased with particle concentration in the water. This is further supported by the lower prevalence of food-like particles in visually oriented fish (see Supplementary Fig. S3 online).

**Table 2:** Results of the generalized regression model (distribution: negative binomial, model fit: elastic net, estimation method: AICc) for visually foraging fish at all time points.

Model effect	Estimates	Number of parameters	Wald chi-square	P-values
Particle appearance	1.21	1	100.42	**<0.0001**
Feeding status	−0.56	1	19.60	**<0.0001**
Particle Concentration	0.00017	1	18.85	**<0.0001**
Fish origin	−0.36	1	8.06	**0.0045**
Particle appearance x particle concentration	0.00015	1	6.83	**0.0090**
Origin x particle concentration	−9.88 × 10^−5^	1	2.75	0.0973

### The role of drinking in the passive uptake of microplastics

**Figure 6:**
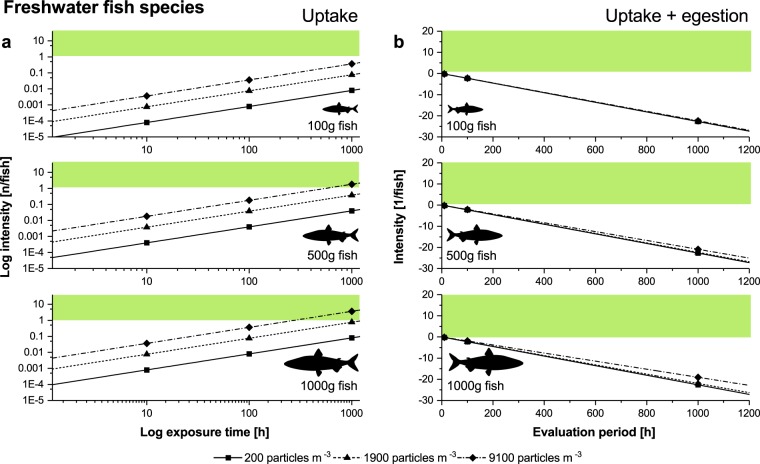
Modelled passive uptake of microplastics via drinking in freshwater fish. **(a)** Uptake rate (coloured area indicates an intensity >1) and **(b)** uptake rate combined with egestion rate for different particle concentrations in the water and fish weights respectively (coloured area indicates an accumulation effect). n = number of particles.

**Figure 7:**
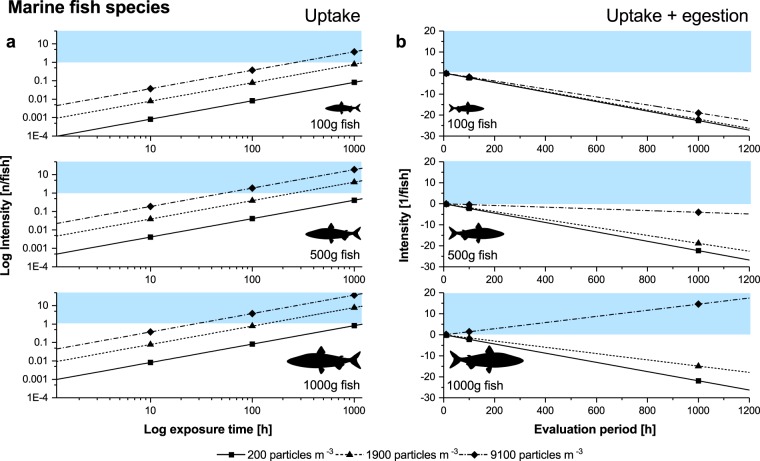
Modelled passive uptake of microplastics via drinking in marine fish. **(a)** Uptake rate (coloured area indicates an intensity >1) and **(b)** uptake rate combined with egestion rate for different particle concentrations in the water and fish weights respectively (coloured area indicates an accumulation effect). n = number of particles.

The results of the modelled uptake and accumulation of microplastic in fish via drinking are shown in Figs. 6 and 7. Independent of fish weight and particle concentration in the water, the probability of freshwater fish ingesting a single microplastic particle is low, occurring approximately once every 300 h in the largest weight class at highest water particle concentrations (Fig. 6(a)). When the egestion of particles is also considered, no accumulation effects were seen to occur (Fig. 6(b)).

In marine fish, the probability of ingesting microplastic particles via drinking is considerably higher at one particle approximately every 30 h in the largest fish weight class at the highest water particle concentrations (Fig. 7(a)). When egestion is considered, no accumulation effects could be observed in the two lower weight classes (Fig. 7(b)), but when looking at the largest fish exposed to highest water particle concentration, an accumulation effect was apparent, with an increase of one particle every 5 days.

**Figure 8:**
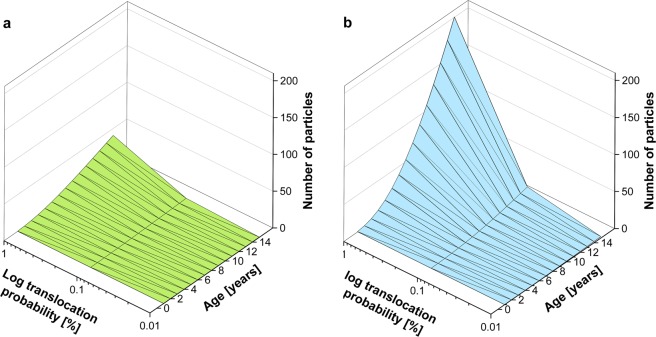
Modelled accumulation of microplastics <5 µm in fish tissue via drinking for **(a)** common carp and **(b)** cod in dependence on hypothesized translocation probability into tissue and age of fish.

To examine the potential long-term accumulation effects of small microplastics in tissues and organs of fish, particle uptake via drinking was modelled exemplarily for common carp (freshwater) and cod (marine). The results are shown in Fig. 8. Assuming that 1% of particles smaller than 5 µm are translocated from chyme into tissue the model predicts that around 40 microplastic particles will accumulate in the tissues of a freshwater common carp over 15 years (Fig. 8(A)). The same assumptions applied to cod, a marine species, suggest that a total of 200 particles will be accumulated (Fig. 8(B)), five times more than in the common carp.

## Discussion

### Biotic factors influencing the microplastic uptake

The detrimental effects of global plastic pollution on aquatic organisms have been extensively studied in recent years^2,4^. In fish, microplastics have been detected in the GIT in marine and freshwater systems alike^22,39,40^. However, the underlying mechanisms of how these particles are ingested are largely unclear. The results of the present study show that several biotic factors play a role. Fish that rely principally on visual foraging cues ingested microplastic particles significantly more often and in higher numbers than species that perform mainly chemosensory foraging, suggesting that a sense of taste aids discrimination of inedible food items. This is further supported by the fact that unlike visual foragers, particle concentration in the water had only a minor effect on the number of particles ingested by chemosensory foraging fish. Nonetheless, some isolated particles were ingested, indicating a potential for accidental uptake. This agrees with laboratory studies using juveniles of the common goby (*Pomatoschistus microps*), which showed an ability to distinguish food and plastic particles, but occasionally still ingested microplastics by mistake^41^.

The profound differences between visual and chemosensory foragers might explain why microplastics are more commonly found in marine pelagic fish species^20,40^. In freshwater systems, the roles of habitat and habitat-related feeding preference are more ambiguous and vary widely between studies^21,22^. It is generally thought that chemosensory foragers feeding near the bottom are more exposed to microplastics, as concentrations are thought to be higher in the sediment than in the water column^31,42^. However, the present results suggest that a developed sense of taste limits the unintentional ingestion of microplastic particles. In the field, other uptake routes, such as transfer via the food chain, might have a more profound effect on the microplastic burden of benthic fish species^13,14^.

It was hypothesized that the genetic origin of fish, be it wild or cultured, has an effect on microplastic uptake. Some studies suggest that selective breeding can result in cultured fish becoming rather undiscerning in their food intake compared to wild individuals^26^. For example, it has been shown that escapees of cultured fish from fish farms have lost their ability to distinguish edible and inedible items^27,28^. No differences were observed in the prevalence of ingested microplastic particles between the wild and cultures groups in this study, despite crucian carp ingesting no microplastics at all. However an abundance trend was apparent, showing that wild fish ingested fewer plastic particles. One factor that weakens the difference between wild and cultured fish might be the large number of wild grayling ingesting microplastics in the present study. The possibility that adaptation to dry pellet food prior to the exposure experiment^43^ or even epigenetic effects belonging to the start period of the laboratory husbandry^44^ had an effect on the ability of fish to differentiate between artificial and real prey cannot be excluded. Further experiments using only natural prey items will have to be conducted to further examine the effect of domestication and husbandry on the propensity for microplastic ingestion.

Finally, active feeding played an important role on the uptake of microplastic particles, where the number of fish ingesting microplastics was higher in tanks where food was being supplied. In contrast, however, the number of particles ingested was higher when no genuine food was available. Thus, feeding behaviour appears to increase the chances of ingesting a microplastic particle, but some fish were actively foraging on microplastics when no food was available. This inappropriate feeding behaviour might be linked to certain particle properties, as discussed below.

### Particle properties influencing microplastic uptake

Previous field studies have shown that some tropical and sub-tropical percid fishes, such as *Girella laevifrons* and *Decapterus muroadsi*, actively ingest microplastics that visually resemble their natural prey^8,9^, and a laboratory study of palm ruff *Seriolella violacea*, showed that particles with similar colour to artificial food pellets were ingested more often^45^. However, it remains unclear if this was a singular case or a common phenomenon among visually foraging fish. Furthermore in this study, only one particle colour was presented at a time with food and only fish that ingested food were considered in the analysis^45^.

In the present study, all particle colours were available simultaneously. Nonetheless, particles with food-like colours were ingested significantly more often than others, independent of whether their colour made them clearly visible or inconspicuous. Interestingly, among wild visually foraging fish, the number of ingested food-like particles increased with increasing concentrations of particles in the water, while there was no increase in the intensity of ingestion of non-food-like colours. This was independent of the availability of genuine food, which again supports the hypothesis that visual foraging fish lack an ability to accurately discriminate edible and inedible food and may even actively forage on microplastics that resembling their prey in terms of colour. With cultured, visually foraging fish, the ingestion of food-like particles also increased with particle concentration in water when food was provided, although to a lesser extent. In the absence of genuine feeding opportunities, both food-like and non-food-like particles were ingested in equal proportions. It is therefore implied that cultured fish did not distinguish between colours and therefore are more prone to active foraging on microplastics regardless of the availability of real food.

A further statistical analysis of the influence of particle colour on microplastic uptake in fish which take a chemosensory approach to foraging was not possible, due to the fact that no plastic particles were detected in crucian carp at all. As already discussed, husbandry effects, i.e. adaption to dry food, might have influenced the tendency to ingest particles in the present exposure experiments. Furthermore, only virgin plastic particles were used in the present exposure experiments. Over time, weathering effects may change the chemical qualities and possibly also the taste of microplastics in the environment, potentially disguising their artificial origin^46^.

Apart from particle colour, particle density also had an effect on the uptake of microplastics. The density of different polymer types dictates whether they float on the water surface or sink to the bottom^47^. Plastic particles with a density greater than water, i.e. sinking particles, were ingested more often than floating particles. This is probably due to the fact that dry pellet food provided during acclimatization and the exposure experiment sank after around 10 seconds to the bottom of the experimental tank (data not shown). Thereby, most active feeding took place at or near the bottom and thus the probability of ingesting sinking plastic particles was increased.

### The role of drinking on the passive uptake of microplastics

The above mentioned uptake routes require that the particle size is large enough to be perceived by fishes as potential food. However recent studies indicate that the majority of microplastics are too small to be detected either by fish or by current monitoring methods^22,48^. These smaller particles may be ingested for other reasons. As shown above, plastic particles can be ingested by accident while foraging or passed on through the food chain^12–14^, but an uptake route which has not yet been looked at is passive or accidental ingestion via drinking, especially in marine fish species^49^. Regular intake of water in marine environments is essential for a number of physiological processes^29^ and marine fish drink about 10 times more water than freshwater fish (see Supplementary Table S1 online). Thus the model predicts low uptake rates in freshwater fish, balanced by frequent egestion which prevents any accumulation of particles. However because of the short duration of the present exposure experiment and the small fish sizes used, it was not possible to fully evaluate the likely effect. In marine fish species, the intake of microplastics via drinking might be more relevant, especially in larger fish, which the model indicates will ingest particles on a regular basis, with a potentially significant accumulation effect when microplastic concentrations in the water are high. Little is known about the microplastic burden in large fishes, due to the limitations of current detection methods^50^. Nonetheless, future research must consider impacts on larger fish and fish species and should include an evaluation of the role of drinking on microplastic burden, not least because of the relevance of many such species as food for human consumption.

Another matter that needs to be considered is the translocation of small microplastics from the GIT into tissues and organs^51,52^. For fishes, information is scarce: there are studies showing the presence of microplastics up to 600 µm in fish liver, but not in muscle tissue^52–54^. On the other hand, laboratory studies by Lu *et al*. 2016^32^ found that only particles smaller than 5 µm were translocated into the liver. This is in accordance with a number of *in vivo* and *in vitro* studies in mammals, showing that translocation rates increase considerably with decreasing particle size^33,34^. The accumulation of microplastics modelled in the present study could show that even when other uptake routes are not considered, and assuming that only 0.01% to 1% of very small particles are regularly translocated, the number of accumulated microplastics might be significant in fishes with a long lifespan, up to 200 particles over 15 years in the modelled cod. The issue is of particular concern for marine species due to their increased water uptake and there are of course important implications for transmission within the food web. The risks of accumulated particles could increase further, if we assume a higher translocation rate of microplastics through the intestinal barrier. Several studies have reported rates over 1% in mammals^34–36^, but rates vary, with particle sizes, properties and experimental setups all seeming to play an important role^55^. Regardless of these uncertainties, there is no information available on how these translocated particles affect might affect fish health and it is not known, if microplastics can be removed from the blood or tissues/organs via natural excretion pathways^56^. It is important that further research considers the uptake of microplastic via drinking, the translocation pathways of micro- and nanoplastics from the GIT of fish, and the potential implications of these phenomena on fish as human food^57^.

## Supplementary information

Supplementary information.

## Data Availability

The datasets generated during and/or analysed during the current study are available from the corresponding author on reasonable request.
