# Supplementary material for: Uptake routes of microplastics in fishes: practical and theoretical approaches to test existing theories
Source: Sci Rep. 2020 Mar 3;10:3896. doi: 10.1038/s41598-020-60630-1 (PMC7054251; doi:10.1038/s41598-020-60630-1)
Supplement: Supplementary file 1 — Supplementary information. [file 41598_2020_60630_MOESM1_ESM.pdf]

Uptake routes of microplastics in fishes: practical and theoretical approaches to test existing theories

S. Roch[1,2*], C. Friedrich[3], A. Brinker[1,2]

[1] Fisheries Research Station Baden-Württemberg, Argenweg 50/1, 88085 Langenargen, Germany. [2] University of Konstanz, Mainaustraße 252, 78464 Konstanz, Germany. [3] FMF Freiburg Material Research Centre and Institute for Macromolecular Chemistry, University of Freiburg, Stefan-Meier-Straße 21, 79104 Freiburg i. Br., Germany

*Correspondance and requests for materials should be addressed to S. R. (email: samuel.roch@lazbw.bwl.de)

Supplementary information

Variable	Habitat	fish size	Value	Reference
Drinking rate	freshwater	20-30 g	0.15 ± 0.03 ml kg^{-1} h^{-1}	Fuentes & Eddy 1997[1]
		20-30 g	0.25 ml kg^{-1} h^{-1}	Fuentes *et al.* 1996[2]
		50 g	1.43 ± 0.31 ml kg^{-1} h^{-1}	Lovegrove & Eddy 1982[3]
		150 - 250 g	0 ml kg^{-1} h^{-1}	Shehadeh & Gordon 1969[4]
		n.a.	0.03 ± 0.01 ml kg^{-1} h^{-1}	Perrot *et al.* 1992[5]
		mean	**0.4 ml kg^{-1} h^{-1}**	
	marine	20-30 g	3.89 ± 0.28 ml kg^{-1} h^{-1}	Fuentes & Eddy 1997[1]
		20-30 g	2.4 ml kg^{-1} h^{-1}	Fuentes *et al.* 1996[2]
		50 g	7.40 ± 1.63 ml kg^{-1} h^{-1}	Lovegrove & Eddy 1982[3]
		150 - 250 g	5.4 ± 0.5 ml kg^{-1} h^{-1}	Shehadeh & Gordon 1969[4]
		n.a.	1.42 ± 0.23 ml kg^{-1} h^{-1}	Perrot *et al.* 1992[5]
		mean	**4.1 ml kg^{-1} h^{-1}**	
Residence time	freshwater, marine	-	51 h	Roch *et al.* (in preparation)
		-	33 h	Grigorakis *et al.* 2017[6]
		-	48 h	Lu *et al.* 2016[7]
		mean	**44 h**	

Table S1. Drinking rates of marine and freshwater fish and residence time of microplastics in the gastrointestinal tract for the model approaches to calculate the passive uptake of microplastics via drinking.

Model	Dependent variable	Fish species	Sampling points	Model effects
Nominal-logistic regression model	prevalence	rainbow trout, grayling, common carp, crucian carp	0 h	origin, foraging style, feeding status, particle concentration, total length, foraging style x feeding
Generalized linear model[a]	abundance	rainbow trout, grayling, common carp, crucian carp	0 h	origin, foraging style, feeding status, particle concentration, total length
	abundance	rainbow trout, grayling, common carp, crucian carp	0 h, 6 h, 24 h	sampling time, fish species, feeding status, particle concentration
	intensity	rainbow trout, grayling, common carp, crucian carp	0 h, 6 h, 24 h	feeding status, particle concentration, particle appearance, particle density
Generalized regression model[b]	intensity	rainbow trout, grayling	0 h, 6 h, 24 h	particle concentration, origin, feeding status, particle appearance, particle appearance x particle concentration, origin x particle concentration

Table S2. Model specifications for each performed model to examine the microplastic uptake of fish in the exposure experiments. [a]A Poisson distribution with a logarithmic link function was assumed for each GLM. Furthermore, an overdispersion parameter was included in each model to adjust variance independent of the mean[8]. [b]A negative binomial distribution was assumed and the model was fitted with help of an elastic net and AIC as the model evaluation method[9].

Fish species	Total length [cm]		Fresh weight [g]	
	mean	SD	mean	SD
rainbow trout	11.3	0.8	17.0	3.8
grayling	11.8	1.0	9.1	2.3
common carp	8.0	0.7	7.3	2.9
crucian carp	7.0	0.5	5.3	1.4

Table S3. Summary of the total length and fresh weight of experimental fish used in the exposure experiments. SD = Standard deviation.

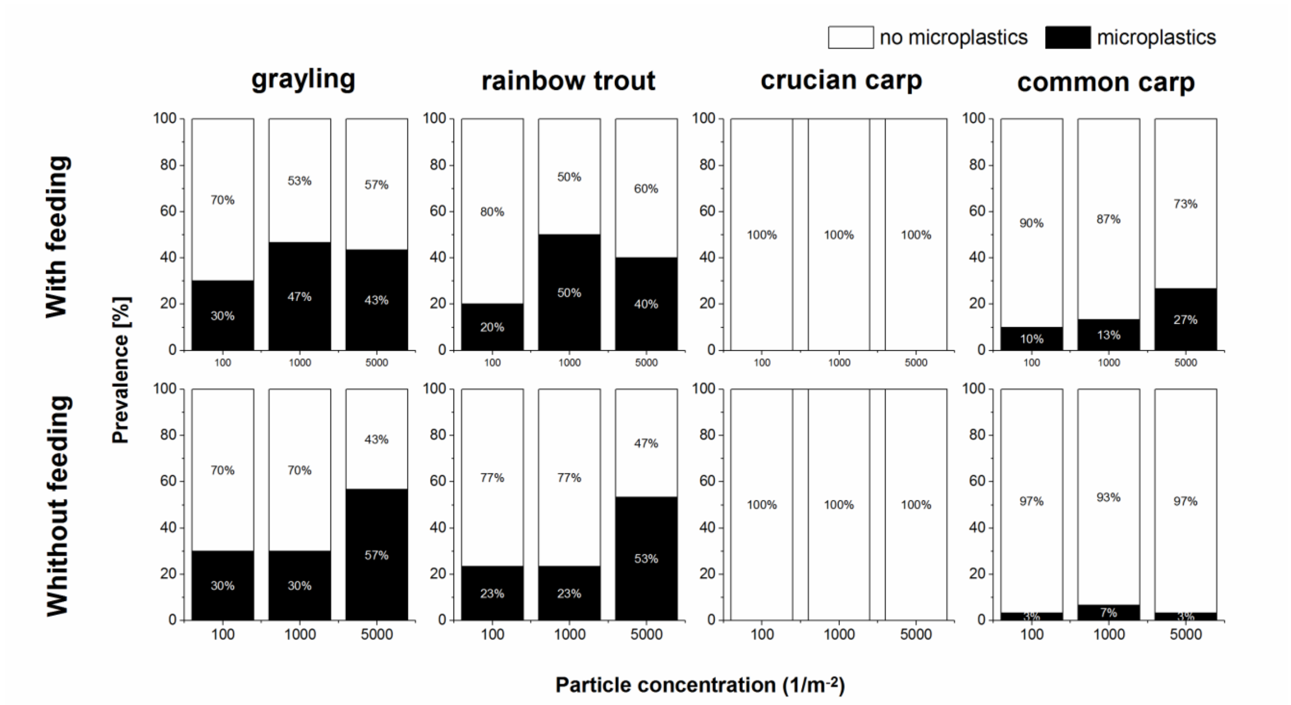

Figure S1. Mean microplastic prevalence for each fish species, particle concentration and feeding status, directly after exposure.

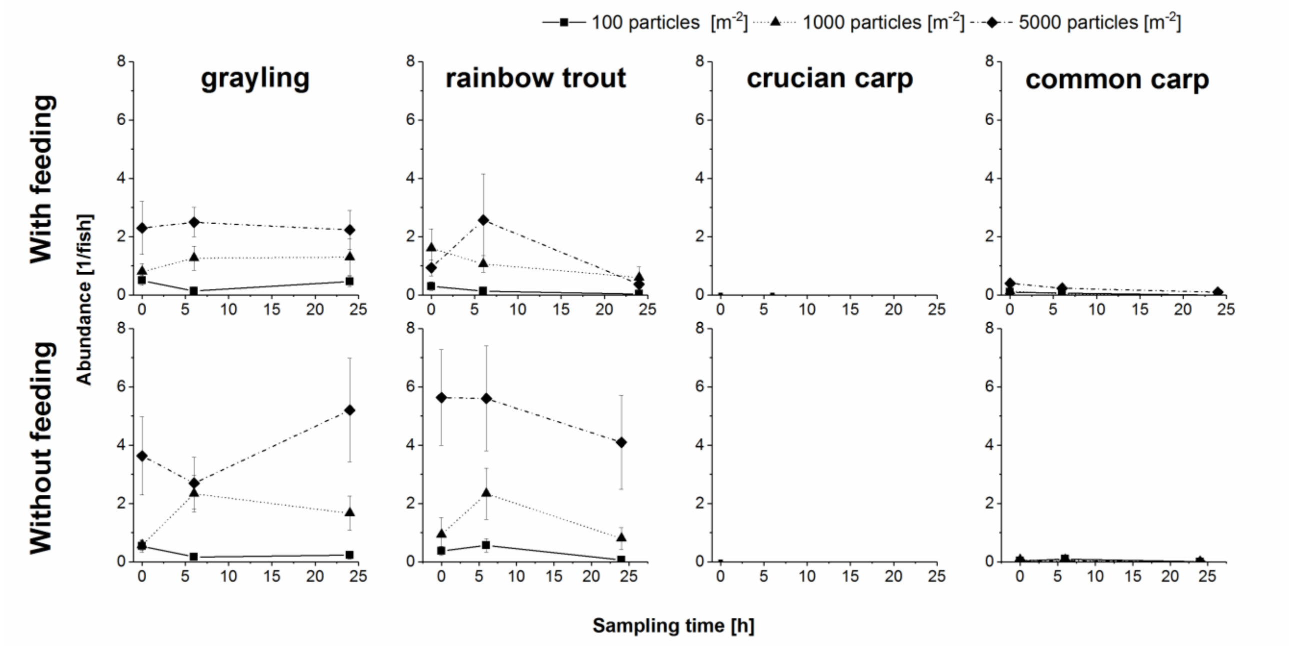

Figure S2. Mean microplastic abundance [± standard error] for each fish species, particle concentration, sampling point and feeding status.

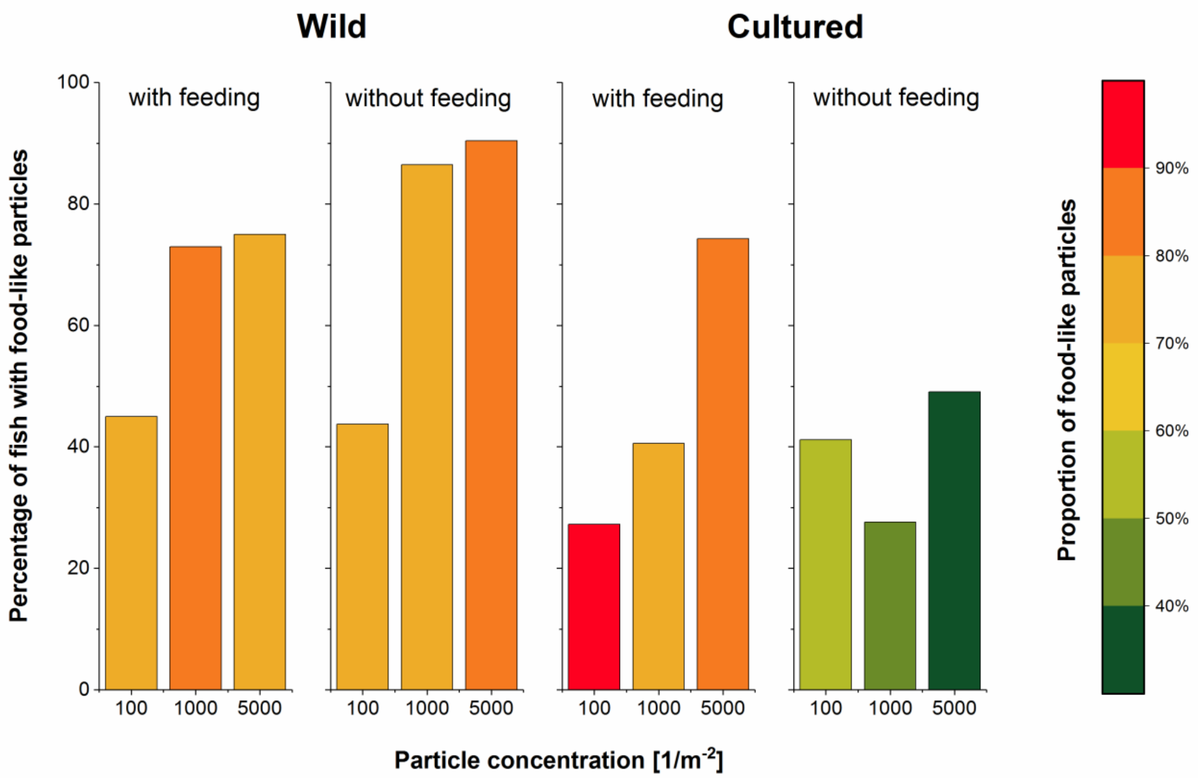

Figure S3. Percentage of fishes with microplastics that ingested at least one food-like particle (all time points). Colours indicate the proportion of food-like particles, compared to all particles ingested.

References

1. Fuentes, J. & Eddy, F. B. Drinking in marine, euryhaline and freshwater teleost fish. in *Ionic Regulation in Animals: A Tribute to Professor W.T.W.Potts* (eds. Hazon, N., Eddy, F. B. & Flik, G.) 135–149 (Springer Berlin Heidelberg, 1997). doi:10.1007/978-3-642-60415-7_9.
2. Fuentes, J., Bury, N. R., Carroll, S. & Eddy, F. B. Drinking in Atlantic salmon presmolts (Sulmo salar L.) and juvenile rainbow trout (Oncorhynchus mykiss Walbaum) in response to cortisol and sea water challenge. *Aquaculture* **141**, 129–137 (1996).
3. Lovegrove, S. M. & Eddy, B. Uptake and accumulation of zinc in juvenile rainbow trout, Salmo gairdneri. *Environ. Biol. Fishes* **7**, 285–289 (1982).
4. Shehadeh, Z. H. & Gordon, M. S. The role of the intestine in salinity adaptation of the rainbow trout, Salmo gairdneri. *Comp. Biochem. Physiol.* **30**, 397–418 (1969).

5. Perrott, M. N., Grierson, C. E., Hazon, N. & Balment, R. J. Drinking behaviour in sea water and fresh water teleosts, the role of the renin-angiotensin system. *Fish Physiol. Biochem.* **10**, 161–168 (1992).

6. Grigorakis, S., Mason, S. A. & Drouillard, K. G. Determination of the gut retention of plastic microbeads and microfibers in goldfish (Carassius auratus). *Chemosphere* **169**, 233–238 (2017).

7. Lu, Y. *et al.* Uptake and Accumulation of Polystyrene Microplastics in Zebrafish (*Danio rerio*) and Toxic Effects in Liver. *Environ. Sci. Technol.* **50**, 4054–4060 (2016).

8. McCullagh, P. & Nelder, J. A. *Generalized Linear Models*. (Chapman and Hall, 1983).

9. Sokal, R. R. & Rohlf, J. F. *Biometry: The Principles and Practice of Statistics in Biological Research*. (W.H. Freemann and Company, 2003).
